# Proceedings of Patient Reported Outcome Measure’s (PROMs) Conference Sheffield 2016: advances in patient reported outcomes research: Sheffield, UK. 9th June 2016

**DOI:** 10.1186/s12955-016-0540-5

**Published:** 2016-10-10

**Authors:** Tim Croudace, John Brazier, Nils Gutacker, Andrew Street, Dan Robotham, Samantha Waterman, Diana Rose, Safarina Satkunanathan, Til Wykes, Nasrin Nasr, Pamela Enderby, Jill Carlton, Donna Rowen, Jackie Elliott, John Brazier, Katherine Stevens, Hasan Basarir, Alex Labeit, Mairead Murphy, Sandra Hollinghurst, Chris Salisbury, Dominic Marley, James Wilson, Amy Barrat, Bibhas Roy, Ines Rombach, Órlaith Burke, Crispin Jenkinson, Alastair Gray, Oliver Rivero-Arias, Ian Porter, Jaheeda Gangannagaripalli, Charlotte Bramwell, Jose M. Valderas, Patricia Holch, Susan Davidson, Jacki Routledge, Ann Henry, Kevin Franks, Alex Gilbert, Kate Absolom, Galina Velikova, Ian Porter, Jose M. Valderas, Jan R. Boehnke, Andrew Trigg, Ruth Howells, Jeshika Singh, Subhash Pokhrel, Louise Longworth, Caroline Potter, Cheryl Hunter, Laura Kelly, Elizabeth Gibbons, Julian Forder, Angela Coulter, Ray Fitzpatrick, Michele Peters

**Affiliations:** 1 University of Dundee, Dundee, UK; 2 University of Sheffield, Sheffield, UK; 3 University of York, York, UK; 4 Kings College London, London, UK; 5 University of Sheffield, Sheffield, UK; 6 University of Sheffield, Sheffield, UK; 7 University of Bristol, Bristol, UK; 8 Central Manchester University Hospitals, Manchester, UK; 9 University of Oxford, Oxford, UK; 10 University of Exeter Medical School, Exeter, UK; 11 University of Leeds, Leeds, UK; 12 University of Exeter Medical School, Exeter, UK; 13 University of York, York, North Yorkshire UK; 14 DRG Abacus, Manchester, UK; 15 Brunel University London, Uxbridge, UK; 16 University of Oxford, Oxford, UK

## Abstract

S1 Using computerized adaptive testing

Tim Croudace

S2 Well-being: what is it, how does it compare to health and what are the implications of using it to inform health policy

John Brazier

O1 “Am I going to get better?”—Using PROMs to inform patients about the likely benefit of surgery

Nils Gutacker, Andrew Street

O2 Identifying Patient Reported Outcome Measures for an electronic Personal Health Record

Dan Robotham, Samantha Waterman, Diana Rose, Safarina Satkunanathan, Til Wykes

O3 Examining the change process over time qualitatively: transformative learning and response shift

Nasrin Nasr, Pamela Enderby

O4 Developing a PROM to evaluate self-management in diabetes (HASMID): giving patients a voice

Jill Carlton, Donna Rowen, Jackie Elliott, John Brazier, Katherine Stevens, Hasan Basarir, Alex Labeit

O5 Development of the Primary Care Outcomes Questionnaire (PCOQ)

Mairead Murphy, Sandra Hollinghurst, Chris Salisbury

O6 Developing the PKEX score- a multimodal assessment tool for patients with shoulder problems

Dominic Marley, James Wilson, Amy Barrat, Bibhas Roy

O7 Applying multiple imputation to multi-item patient reported outcome measures: advantages and disadvantages of imputing at the item, sub-scale or score level

Ines Rombach, Órlaith Burke, Crispin Jenkinson, Alastair Gray, Oliver Rivero-Arias

O8 Integrating Patient Reported Outcome Measures (PROMs) into routine primary care for patients with multimorbidity: a feasibility study

Ian Porter, Jaheeda Gangannagaripalli, Charlotte Bramwell, Jose M. Valderas

O9 eRAPID: electronic self-report and management of adverse-events for pelvic radiotherapy (RT) patients

Patricia Holch, Susan Davidson, Jacki Routledge, Ann Henry, Kevin Franks, Alex Gilbert, Kate Absolom & Galina Velikova

O10 Patient reported outcomes (PROMs) based recommendation in clinical guidance for the management of chronic conditions in the United Kingdom

Ian Porter, Jose M.Valderas

O11 Cross-sectional and longitudinal parameter shifts in epidemiological data: measurement invariance and response shifts in cohort and survey data describing the UK’s Quality of Life

Jan R. Boehnke

O12 Patient-reported outcomes within health technology decision making: current status and implications for future policy

Andrew Trigg, Ruth Howells

O13 Can social care needs and well-being be explained by the EQ-5D? Analysis of Health Survey for England dataset

Jeshika Singh, Subhash Pokhrel, Louise Longworth

O14 Where patients and policy meet: exploring individual-level use of the Long-Term Conditions Questionnaire (LTCQ)

Caroline Potter, Cheryl Hunter, Laura Kelly, Elizabeth Gibbons, Julian Forder, Angela Coulter, Ray Fitzpatrick, Michele Peters

## S1 Using computerized adaptive testing

### Tim Croudace (t.j.croudace@dundee.ac.uk)

#### University of Dundee, Dundee, UK

Health, health-related and health-care evaluation research can consider technologies that are adaptive in some way: this conference focuses interest on Patient Reported Outcome Measures but arguably its remit could be widened. Why? Statistical methodology, health economics and health psychometrics are seen to converge in use of models and understanding of multiple item, multi-construct or multidimensional questionnaire (or test) data on occasion.

Fieldwork in health and related research using legacy instruments (tests and scales) is a massive undertaking and social survey, cohort and large scale longitudinal research in biomedicine have progressed towards platform scale. Innovation using computer-based or computerized adaptive tests is relatively new in the UK in some sectors and for some scientific communities, but this scientific meeting will provide valuable updates and visibility to ongoing work.

Extensions to existing evaluation data are easily motivated and you are welcomed to join this initiative and conversation with evaluation models, new domains of application for simple developments as well as more complex and challenging applications.

Healthcare technology cooperatives and health data analytics networks are logical places to progress relevant work and collaborations but – if warranted – sustainable development of evaluation at scale requires platforms accessible but not necessarily linked between cognitive and non-cognitive outcomes, health and social care, PROMS and epidemiological measures. In my opinion (dynamically updated!) CAT and variants are in their infancy in NIHR research and CLAHRC collaboration could offer leadership in this area (I can speak to some possible contributions).

Research council, charity and industry sector collaboration might be entertained as in other areas of applied science. Strategic investment in coordinated activity supporting feasibility evaluation and development at scale in the NHS or other organisations relevant to health or related research is needed to accelerate and extend existing programmatic activity, though incremental progress is being made by pioneers and early adopters (though the field is decades old). Options for one country, organisation, setting or collaborator might not all be the same, but sharing of potentials and early evaluation results is encouraged since this might allow new partners to engage and progress. Interest from professional groups (whether clinical area, education sector (medical, dentistry, nursing and allied health professional groups) is increasing and may promote participation in platform style developments in coming years. Relationships with internationally visible initiatives and platform developments remain as opportunities.

## S2 Well-being: what is it, how does it compare to health and what are the implications of using it to inform health policy

### John Brazier

#### University of Sheffield, Sheffield, UK

There has been a growing interest in using measures of subjective well-being to inform decision making within Government departments (such as health) and between them (such as between health and social care) (e.g. Legatum report). It has been suggested that measures of subjective well-being like life satisfaction and happiness would provide a broader measure of benefit than health, and furthermore they would enhance the consistency and comparability between Government departments, and could be used to improve the efficiency and equity in the use of scarce public resources. However, there are concerns about the relevance and suitability of subjective well-being measures in the context of health.

This paper examines the different conceptual accounts of well-being and how these compare with the empirical evidence using factor analyses. It then compares different instruments for measuring subjective well-being with each other and with health to better understand their sensitivity to health conditions using four data sets (USoC, HSE, HoDAR, and MIC) both cross-sectionally and longitudinally (USoC only). Finally, it considers how well-being could be incorporated into economic evaluation to evaluate health and social care policies (e.g. through an adapted QALY). It concludes that greater reliance on well-being to measure the benefits of health care would have radical implications for the priorities of the NHS.

## O1 “Am I going to get better?” - Using PROMs to inform patients about the likely benefit of surgery

### Nils Gutacker, Andrew Street

#### University of York, York, UK

##### **Correspondence:** Nils Gutacker – University of York, York, UK

The English NHS is the first healthcare system to mandate the routine collection of patient-reported outcome measures (PROMs) after routine surgery. One aim of this policy is to provide patients and their general practitioners (GPs) with information on the quality of local health services and the likely benefit of surgery. Yet, there is concern that these data remain under-utilised; in part because they are difficult to access and interpret. Anecdotal evidence from GPs in the Vale of York suggests that PROM data can play an important part in patient discussions with their GPs, but only if these data are presented in a meaningful way and in lay friendly formats.

To develop a web tool for use by patients and their GPs to predict their post-operative health status and present this in a simple and visually appealing way.

Anonymised patient-level EQ-5D data for over 260,000 NHS patients who underwent hip or knee replacement surgery or groin hernia repair between April 2009 and March 2015 were obtained from the HSCIC. Classification tree analysis was used to develop algorithms that predict post-operative EQ-5D utility scores based on age, gender and pre-operative EQ-5D profiles. For each EQ-5D dimension results were presented as proportions of ‘patients like you’ that felt noticeably better/not different/worse, based on estimates of the minimally important difference. Results were also presented as proportions of patients reporting no/some/extreme problems post-operatively. Proportions were expressed as green/yellow/red circles to facilitate interpretation.

Patients were classified into 44 (hernia repair) to 56 (knee replacement) groups. The proportion of variance explained ranged from 14 % to 27 %.

It is feasible to develop a web tool to inform patients about their likely surgical outcomes given their pre-operative characteristics.

## O2 Identifying Patient Reported Outcome Measures for an electronic Personal Health Record

### Dan Robotham, Samantha Waterman, Diana Rose, Safarina Satkunanathan, Til Wykes

#### Kings College London, London, UK

##### **Correspondence:** Dan Robotham – Kings College London, London, UK

We sought stakeholder feedback on various self-completed Patient Reported Outcome Measures (PROMs) commonly used in clinical practice. We aimed to make the most appropriate PROMs available to mental health service users within an electronic Personal Health Record (ePHR).

An initial consultation with service users and clinicians suggested that ‘mood’ and ‘worry’ were important outcomes to measure. We reviewed the available PROMs for these outcomes, selecting those which were valid, sensitive to change, free of copyright and considered useful to clinicians.

We used a mixture of nominal and Delphi group techniques to gain a consensus from stakeholders. This included two nominal groups with 19 service users in total, one nominal group with ten Allied Health Professionals (AHPs), and one online Delphi group with eight consultant psychiatrists. In nominal groups, participants were asked to review PROMs relating to mood and worry, rate them in terms of appropriateness for the task, share average ratings with the group, engage in group discussion, then re-rate following discussion. Similar processes were adopted for the Delphi group, without a group discussion.

The Warwick Edinburgh Mental Wellbeing Scale (WEMWBS) was the most popular PROM for measuring mood. However, concerns were expressed by some service users because it posed questions about the future, which may be unhelpful. Psychiatrists liked the Public Health Questionnaire (PHQ9) and the WEMWBS, but suggested that additional PROMs on high mood/elation should be included. For measuring worry, the Generalised Anxiety Disorder scale (GAD7) was the most popular among all groups.

The WEMWBS, PHQ9 and GAD7 comprise a basic set of PROMs through which service users can self-monitor symptoms of mood and worry within an ePHR. Including a questionnaire on high mood states may be helpful, such as the Goldberg Mania questionnaire.

## O3 Examining the change process over time qualitatively: transformative learning and response shift

### Nasrin Nasr, Pamela Enderby

#### University of Sheffield, Sheffield, UK

##### **Correspondence:** Nasrin Nasr – University of Sheffield, Sheffield, UK

The experience of living with a long-term condition transforms and change individuals. This change process is referred to as transformative learning in the adult learning education (Mezirow 1991) and response shift in health-related quality of life literature (Sprangers & Schwartz, 1999).

In this paper, we draw on the theories underpinning these concepts to demonstrate the need for qualitative approaches to investigate the change process over time when applying PROMs.

We have examined three main theories and their related literature: 1) Transformative learning (Mezirow 1991, Dubouloz 2004), 2) the modified model of coping theory (Folkman 1997), 3) and the narrative model of time and the concept of redefinition of life experience (Mishler 2006).

1. Transformative learning is defined as a “process involving a deconstruction and reconstruction of meaning perspectives”, which will result in a restructuring of the experience and creating a new self-identity.
2. According to the modified model of coping theory, people constantly appraise and reappraise the events through goal processes. During adaptive goal processes people modify their beliefs and expectations and generate new goals as their previous goals are no longer tenable. However, maladaptive goal processes occur when people stay focus on their respected goals despite the fact that they are not attainable any more.
3. The narrative model of time allows individuals to make sense of their past experiences in the light of their later circumstances. Through this event, they change their understanding of self and create a new identity for themselves.

Educational, psychological and sociological theories show that over the course of a long-term condition people change their perception. The notion of reinterpretation of life as examined in three disciplines and by qualitative approaches has great impact on clinical outcomes and illness management when applying PROMs over time.

## O4 Developing a PROM to evaluate self-management in diabetes (HASMID): giving patients a voice

### Jill Carlton, Donna Rowen, Jackie Elliott, John Brazier, Katherine Stevens, Hasan Basarir, Alex Labeit

#### University of Sheffield, Sheffield, UK

##### **Correspondence:** Jill Carlton – University of Sheffield, Sheffield, UK

To develop a PROM to evaluate self-management of diabetes mellitus (DM). This was part of a larger project valuing the non-health consequences of self-management of diabetes to inform health technology assessment of self-management interventions.

Stage 1 identified the attributes of self-management using 5 steps. First a literature review was undertaken to identify and understand themes relating to self-management of DM to inform a topic guide. Second the topic guide was further refined following consultation with a Patient and Public Involvement group. Third the topic guide was used to inform semi-structured interviews with patients with DM to identify how self-management of DM affects individuals. Fourth the research team considered potential attributes alongside health attributes from an existing measure to produce an instrument reflecting both health and self-management outcomes simultaneously. Finally a draft instrument was tested in a focus group to determine the wording and acceptability. In Stage 2 the PROM was administered to individuals with diabetes and the general population in an online discrete choice experiment survey. Classical psychometric analysis was conducted on the data.

Semi-structured interviews were carried out with 32 patients. Eight potential attributes were identified: fear/worry/anxiety; guilt; stress; stigma; hassle; control; freedom; and feeling supported. Four self-management attributes were selected with four health attributes to be used in the online survey in Stage 2 (the HASMID questionnaire). Initial psychometric analysis indicates that HASMID demonstrates good reliability (Cronbach’s α = 0.79) and validity. HASMID could detect differences between the general population and DM sample (M = 16.6, SD = 4.2: M = 15.2, SD = 4.2; t(3741) = 8.4, p < 0.05), and between type 1 and type 2 DM groups (M = 13.9, SD = 4.2: M = 16.6, SD = 3.8; t(751) = −9.0, p < 0.05).

HASMID is a short questionnaire (eight items each with four response levels) with high content and face validity, which is able to evaluate self-management in DM. Further psychometric analysis is currently being undertaken to explore the relationships between some items.

## O5 Development of the Primary Care Outcomes Questionnaire (PCOQ)

### Mairead Murphy, Sandra Hollinghurst, Chris Salisbury

#### University of Bristol, Bristol, UK

##### **Correspondence:** Mairead Murphy – University of Bristol, Bristol, UK

As the first contact for any health-related need, primary care clinicians often address multiple patient problems, with a range of possible outcomes. There is currently no patient-reported outcome measure (PROM) which covers this range of outcomes. This abstract describes the quantitative testing of the Primary Care Outcomes Questionnaire (PCOQ) a 27-item questionnaire designed to capture outcomes which patient seek and primary care clinicians can influence.

Patients completed the PCOQ in GP waiting rooms before a consultation, taking a second questionnaire which contained the PCOQ and validated comparator PROMs for completion after 1 week. The data analysis included:

1. Factor analysis on the PCOQ questionnaires and calculation of factor scores.
2. Linear regression and correlation coefficients between the factor scores and the comparator questionnaires.
3. Effect sizes of the change in PCOQ factor scores.

Six hundred two patients completed the PCOQ in GP waiting rooms, and 264 of these returned the second set of questionnaires. Exploratory factor analysis on the 602 PCOQ questionnaires revealed 4 dimensions:

1. Health Status: physical and emotional symptoms, life effects and health concerns.
2. Health Knowledge and Self-Care: understanding of health problems, ability to stay healthy, manage symptoms and prevent future health problems.
3. Confidence in Health Plan: confidence in and adherence to health plans.
4. Confidence in Health Provision: confidence in accessing appropriate primary healthcare when needed.

Each dimension was associated as expected with respective comparator PROMs. The sub-set of patients who said their main problem had improved had small to moderate effect sizes for each construct.

The PCOQ was acceptable to patients and easily administered in GP waiting rooms. It showed a clear factor structure and evidence for construct validity, including responsiveness to change. It is a promising new tool to assess the outcome of primary care interventions from a patient perspective.

## O6 Developing the PKEX score- a multimodal assessment tool for patients with shoulder problems

### Dominic Marley, James Wilson, Amy Barrat, Bibhas Roy

#### Central Manchester University Hospitals, Manchester, UK

##### **Correspondence:** Dominic Marley – Central Manchester University Hospitals, Manchester, UK

We aimed to develop a new multi-modal shoulder score that incorporated different outcome domains. To ensure the score did not become clinician reported, assessment of shoulder range of motion would be monitored using automated sensors connected to a laptop.

Using a multidisciplinary team composed of consultants, trainees, nurses and physiotherapists we identified four key areas that are central to patient’s outcomes. We created ‘PKEX’ a composite outcome comprised of patient reported outcomes (P), kinematics of the joint (K), patient Engagement with the rehabilitation process (E) and the patient’s experience of treatment (X). A bank of questions was developed for PROMs and patient experience using themes from patients, surgeons and current scoring systems (OSS, DASH). Over 12 weeks patients with shoulder problems were invited to attend focus groups to identify the most important questions related to their shoulder. The kinematic component was assessed using automated sensor based technology and the system was rated using the Systems Usability Score (SUS) completed by patients.

70 patients attended the focus groups. Patients attending ranged from new outpatient attendees to those who had been discharged to virtual follow-up clinics.

Common themes identified for PROMs included pain levels, ability to function and the need for analgesia. Patient experience themes centered on the transfer of information, shared decision making and the quality of care received.

Patients responded positively to sensor based technology in assessing shoulder range of motion. The usability of the system was scored at 74 % on the SUS.

Further work is required to validate the individual components of the PKEX score. Ethical approval has been granted to assess PKEX further as part of a randomised control trial. The PKEX score may allow the patient and clinician to have a more comprehensive understanding of their post-intervention outcome.

## O7 Applying multiple imputation to multi-item patient reported outcome measures: advantages and disadvantages of imputing at the item, sub-scale or score level

### Ines Rombach, Órlaith Burke, Crispin Jenkinson, Alastair Gray, Oliver Rivero-Arias

#### University of Oxford, Oxford, UK

##### **Correspondence:** Ines Rombach – University of Oxford, Oxford, UK

Missing data are generally unavoidable in clinical trials (RCTs), particularly in patient reported outcome measures (PROMs) and can introduce bias into the study results. Multiple imputation (MI) is considered to be one of the most reliable methods to handle this problem.

Traditionally applied to the full PROMs score of multi-item instruments, some recent research suggests that MI at the item level may be preferable under certain scenarios.

We present practical guidance on the choice of MI models, and offer advice on improving convergence of complex models.

We simulated missing at random data based on a previously published algorithm within the follow-up data from an RCT trial using three different PROMs (OKS, EQ-5D-3 L, SF-12). Simulated datasets had 100–1000 observations and 5 %–40 % of missing outcome data.

Data was multiply imputed at the item, sub-scale and score level; treatment coefficients from linear regression models were obtained for 1000 simulations. Mean absolute errors (MAE) were used as performance parameters.

Good convergence for item-level MI was observed for samples of 1000 and 5 % of missing data. Non-convergence increased to 19 % (EQ-5D-3 L), 28 % (OKS) and 80 % (SF-12) for 40 % of missing data and 89 % (OKS) for sample sizes of 100.

For the OKS, a MAE in the treatment coefficient of around 0.12 was observed for imputations at the item and score level for 5 % of missing data and 1030 observations. The MAE increases to 0.47 (sore MI) and 0.44 (item MI) for 40 % of missing data, and 0.43 (score MI) and 0.40 (item MI) for a sample size of 100.

Small changes to the default imputation code, re-categorisation of categorical data and simplification of the MI model can improve convergence.

Overall, the MAE decreases as sample size increases, as well as with decreasing overall levels of missing data. Imputation at the score and subscale level outperforms imputation at the item level in small sample sizes. All approaches provide similar results for large sample sizes and low levels of missing data.

## O8 Integrating Patient Reported Outcome Measures (PROMs) into routine primary care for patients with multimorbidity: a feasibility study

### Ian Porter, Jaheeda Gangannagaripalli, Charlotte Bramwell, Jose M. Valderas

#### University of Exeter Medical School, Exeter, UK

##### **Correspondence:** Ian Porter – University of Exeter Medical School, Exeter, UK

Patient Reported Outcome Measures (PROMs) are currently under-utilised in Primary Care. For patients with multiple conditions (multimorbidity), combinations of individualised and standardised PROMs may support health management and prioritisation. We aimed to test the feasibility of implementing routine PROMs administration and feedback as part of Primary Care annual reviews for patients with multimorbidity.

Patients with two or more chronic conditions (asthma, COPD, diabetes, heart failure, depression, and osteoarthritis of the hip and/or knee) completed generic, condition specific and individualised PROMs immediately ahead of annual reviews. Personalised PROM summaries were provided to patients and clinicians to inform reviews. Acceptability of the intervention was rated by patients/clinicians separately. Qualitative interviews were also conducted with patients (10) and clinicians (5) to provide a more detailed evaluation.

All 68 recruited patients (mean age 70; 47 % female) completed the relevant PROM measures, and received personalised feedback, ahead of their review. The most common combinations of conditions were diabetes/COPD (18 % of participants) and diabetes/osteoarthritis of the knee (13 % of participants). 90 % of patients agreed/strongly agreed that the PROMs summary had been a useful way of facilitating health prioritisation. Clinicians agreed/strongly agreed that the feedback was helpful for conducting the review for 89 % of patients. Analysis from qualitative interviews indicated that both patients and clinicians viewed the PROMs review positively, considering it to be comprehensive and patient centred.

This is the first evaluation of the role of routine PROMs assessment of patients with multimorbidity in Primary Care. Preliminary findings suggest a high degree of acceptability from both patients and clinicians. Based on identified barriers and facilitators, recruitment pathways, research instruments, and administration methods will be refined with the aim of conducting a full scale randomised evaluation of this intervention.

## O9 eRAPID: electronic self-report and management of adverse-events for pelvic radiotherapy (RT) patients

### Patricia Holch, Susan Davidson, Jacki Routledge, Ann Henry, Kevin Franks, Alex Gilbert, Kate Absolom & Galina Velikova

#### University of Leeds, Leeds, UK

##### **Correspondence:** Patricia Holch – University of Leeds, Leeds, UK

Patients undergoing pelvic RT undergo both acute and long term AE (up to 20 % gastrointestinal problems, and 30–45 % post-RT sexual dysfunction). Adverse events (AE) are rarely systematically recorded and often under-reported in clinical practice. A feasible cost-effective model to allow remote measurement of RT AE is required. eRAPID is a web-based electronic patient reporting system including severity linked alerts/self-management advice 1,2,3. (an RCT assessing feasibility in systemic therapy is underway). An eRAPID system for patients undergoing pelvic RT is being developed in St James’s Institute of Oncology-Leeds and the-Christie Hospital Manchester.

To develop eRAPID for (prostate, anal/rectal, gynaecological) RT patients we will 1) identify and develop appropriate PROs (patient-reported-outcomes) to facilitate remote symptom reporting, 2) develop and display self-management advice for low level problems and determine severity related algorithmic treatment responses, 3) map the process of current treatment pathways via interviews with patients, key professionals and carers, 4) successfully integrate PRO questionnaire software (QTool) into existing electronic patient records and RT delivery systems to facilitate ‘real time’ data flow.

We have selected appropriate validated PRO AE measures from systematic reviews 4,5,6. Self-management advice has been developed for low level AE (≤CTCAE grade 2). We have successfully mapped the patient pathways and identified the key health professionals placed to introduce the eRAPID system. We will assess feasibility in RT in a multi-site pilot study (N = 168).

We envisage eRAPID will bring benefits for patients (better self-management of mild AE, earlier detection/treatment of late AE, increased patient confidence), benefits for clinicians (improved AE documentation, patient management and audit) and benefits to the health service (reducing costs from hospital contacts/admissions). Ultimately collection of ePRO AE will allow the development of predictive models of care and comparison and evaluation of new RT approaches.

## O10 Patient reported outcomes (PROMs) based recommendation in clinical guidance for the management of chronic conditions in the United Kingdom

### Ian Porter, Jose M.Valderas

#### University of Exeter Medical School, Exeter, UK

##### **Correspondence:** Ian Porter – University of Exeter Medical School, Exeter, UK

This aim of this project was to investigate what clinical guidance is available in the UK linking patient reported outcome (PRO) measurements to the management of chronic conditions.

Six chronic conditions were selected covering a wide range of impacts on symptoms and functioning, comprising both physical and mental health, and clinical progression: asthma, COPD, diabetes, heart failure, depression, and osteoarthritis (hip and/or knee). All available guidance (clinical practice guidelines and quality indicators) in the NICE portal was systematically searched. Data extracted verbatim included: the type of information available, named PROM instruments (if available) and whether recommendations were explicit in linking PROM scores to the management of conditions.

The PRO construct identified included symptoms, functional status, and health related quality of life. Potentially relevant PROMs were identified for all conditions (ranging from 3 to 7), except for diabetes and heart failure. The most frequent recommendation for the use of PROMs was for assessing the clinical status of patients (all, except for diabetes and heart failure) and there were further recommendations for informing and evaluating treatment in depression and osteoarthritis. Interventions for improving PROs were specifically identified for asthma, COPD and osteoarthritis. COPD was an exception in that PROM scores (MRC dyspnoea scale) were explicitly linked to management options.

There is limited information available in current UK clinical guidance linking PROM information with specific advice for the management of the six selected chronic conditions. Although a number of named PROMs have been identified, recommendations in relation to their use for informing and evaluating treatment are both infrequent and non-specific. This represents a significant barrier to PROMs becoming a routine part of Primary Care in the UK.

## O11 Cross-sectional and longitudinal parameter shifts in epidemiological data: measurement invariance and response shifts in cohort and survey data describing the UK’s Quality of Life

### Jan R. Boehnke

#### University of York, York, North Yorkshire, UK

Archival survey data on Quality of Life (QoL) allows epidemiologists to produce adequate references to inform clinical research. In the UK, numerous cross-sectional and longitudinal surveys have been covering aspects of QoL for more than twenty years and this presentation reports on ongoing efforts to link these data and to build a comprehensive reference data base of the UK population’s QoL.

The outcome across studies for this reference case is the General Health Questionnaire (Goldberg et al., 1988, GHQ-12), assessed in the Health Survey for England (cross-sectional; individual household respondents 2005: N = 6157; 2006: N = 8197) and the British Household Panel Survey (longitudinal; individual household respondents 2005 and 2006 N = 4779). Different parameterisations of the Rasch Model and a set of categorical data structural equation models are used to investigate measurement invariance and response shifts and to produce commonly anchored estimates of QoL.

In this reference scenario the GHQ-12 is shown to be largely measurement invariant as well as free of response shifts. The presentation compares the consequences of using different models for individual estimates as well as area estimates for government regions. Especially the latter case shows the value of the integration of multiple data sets, since it increases the sample size when many groups need to be considered in testing for measurement invariance.

Integrating large data sets from different sources allows widening the coverage of populations and broadening time horizons. While standard approaches exist for data integration, establishing the same metric across time, studies, and (potentially) instrument combinations is for more challenging. Two challenges will be highlighted in the discussion: (a) the need for effect size measures to decide on the relevance of effects; and (b) establishing longitudinal and cross-sectional measurement invariance when combining data from both design types.

## O12 Patient-reported outcomes within health technology decision making: current status and implications for future policy

### Andrew Trigg, Ruth Howells

#### DRG Abacus, Manchester, UK

##### **Correspondence:** Andrew Trigg – DRG Abacus, Manchester, UK

In contrast to regulatory settings, research on using patient-reported outcome (PRO) data within health technology assessment (HTA) is limited. The objectives of this research were to: 1) document PRO-related guidance issued by the National Institute for Health and Care Excellence (NICE); 2) explore manufacturers’ compliance with this; 3) understand NICE’s acceptance of deviations from the guidance; and 4) identify areas of improvement within this process.

After identifying PRO-related guidance from NICE, documentation on new single technology appraisals (STAs) published throughout 2014 was reviewed to identify PRO data usage and its compliance with NICE’s guidance. Reviews of existing STAs, and medical device STAs were excluded. PRO data supporting cost-effectiveness and clinical-effectiveness was explored.

NICE published new STA guidance on 19 pharmaceutical products throughout 2014; 16 documented a recommendation. Regarding cost-effectiveness, PRO approaches mostly adhered to NICE PRO-related guidance, with 84 % of STA submissions measuring health-related quality of life (HRQoL) using the EQ-5D. However, transparency regarding the valuation of HRQoL appears to be lacking; 47 % of submissions did not provide the valuation method. Over half (58 %) of STA guidance documents cited PRO data to support clinical-effectiveness. Despite NICE guidance on outcome measures used to support clinical-effectiveness (requesting evidence of reliability or validity) this was mentioned by the manufacturer for less than a quarter (22 %) of the measures used. Interestingly, neither the evidence review group nor committee seemed to comment regarding this disconnect.

Although PRO data’s role in supporting cost-effectiveness is clearly guided and adhered to, transparency issues remain. In contrast, clinical-effectiveness guidance is vague and compliance is low which appears to be currently unrecognised as an issue. Therefore, a more stringent approach is needed when assessing PRO data within HTA, to ensure accurate measurement of treatment effectiveness to inform better decision making.

## O13 Can social care needs and well-being be explained by the EQ-5D? Analysis of Health Survey for England dataset

### Jeshika Singh, Subhash Pokhrel, Louise Longworth

#### Brunel University London, Uxbridge, UK

##### **Correspondence:** Jeshika Singh – Brunel University London, Uxbridge, UK

The recent shift to an integrated approach to health and social care enables cohesive support to those who are in need of care, but raises a challenge in terms of comparing the diverse benefits from different types of care in order to inform resource-allocation decisions across the two sectors. EQ-5D is the most commonly used measure of health-related quality of life but there is currently little clarity about how it is associated with other measures used in social care. This study will investigate the relationship between health and wellbeing measures and social care needs in a bid to understand the relationship between the measures.

We empirically compared responses to health and wellbeing measures and social care needs from a large cross-sectional dataset of the general population called the Health Survey for England. Multivariate analysis was carried out to examine whether social care needs measured by the Barthel Index can be explained by health status as captured by EQ-5D and wellbeing measures WEMWBS and GHQ-12.

Our study found that poor overall score in EQ-VAS, EQ-5D Index, GHQ-12 and WEMWBS indicated a need for social care. Investigation of the dimensions found that EQ-5D dimensions: self-care and pain/discomfort - were significantly associated with need for social care. In addition, individuals with extreme anxiety/depression (compared to those without) and unable to perform usual activities (compared to those with no problems) were more likely to report need for social care. None of the GHQ-12 dimensions and two dimensions from WEMWBS, ‘been feeling useful’ and ‘had energy to spare,’ was significantly associated with the Barthel index.

The results show that the need for social care, which is dependent on ability to perform personal day-to-day activities, is more closely related to the EQ-5D dimensions which assess physical and mental health, than wellbeing measures WEMWBS and GHQ-12.

## O14 Where patients and policy meet: exploring individual-level use of the Long-Term Conditions Questionnaire (LTCQ)

### Caroline Potter, Cheryl Hunter, Laura Kelly, Elizabeth Gibbons, Julian Forder, Angela Coulter, Ray Fitzpatrick, Michele Peters

#### University of Oxford, Oxford, UK

##### **Correspondence:** Caroline Potter – University of Oxford, Oxford, UK

This paper explores how patients (rather than researchers or clinicians) might engage with a new PROM, the Long-Term Conditions Questionnaire (LTCQ). Recognizing the current health and social care policy emphasis on measuring patient-reported outcomes, we consider how outcomes can be assessed through the routine individual-level use of PROMs.

Forty-two participants with a wide range of long-term conditions (LTCs), including physical and mental health and multi-morbidity, were recruited through primary care. Semi-structured interviews were audio-recorded, transcribed, and analysed using NVivo. Data coded under the theme ‘value of a long-term conditions questionnaire’, which focused on whether information captured in a PROM for LTCs would be useful to patients, are presented here.

Participants indicated that a PROM for LTCs would be most useful to them as a tool for informing individual-level care. They signalled the PROM’s value as a ‘conversation starter’ with health and social care professionals, as a means of prompting and structuring discussion on issues that concerned them. Some participants also indicated its value for capturing changes in their health status over time, potentially opening dialogue with health professionals across multiple services about unmet need.

Monitoring quality of life for people with LTCs currently occurs only at population level, for example through the GP Patient Survey or the Adult Social Care Survey. Patients’ perceived value of the LTCQ aligns with aims to extend the use of patient-reported outcome measures for informing individual-level care. As a PROM that taps into broad domains of living well with LTCs, the LTCQ could be used by individual patients to facilitate routine health reviews and communication across multiple services. The LTCQ could thus play a role in operationalising current policy goals, such as enhancing personalised care planning and encouraging coordination across health and social care.

